# 易栓症诊断与防治中国指南（2021年版）

**DOI:** 10.3760/cma.j.issn.0253-2727.2021.11.001

**Published:** 2021-11

**Authors:** 中华医学会血液学分会血栓与止血学组

易栓症（thrombophilia）是指因各种遗传性或获得性因素导致容易发生血栓形成和血栓栓塞的病理状态[1]。易栓症的主要临床表现为静脉血栓栓塞症（venous thromboembolism, VTE）：如深静脉血栓形成、肺栓塞、颅内静脉血栓形成、门静脉血栓形成、肠系膜静脉血栓形成等；某些类型的易栓症可表现为年轻早发的急性冠脉综合征、缺血性卒中等动脉血栓事件[2]。易栓症导致的血栓事件反复发作显著增加了患者的致残率和致死率，严重危害人类健康。为规范我国易栓症的诊治，经中华医学会血液学分会血栓与止血学组专家讨论，结合GRADE（Grade of Recommendations Assessment, Development, and Evaluation）分级系统——即证据等级和推荐程度的评估[3]而制订本指南。

一、易栓症的发病机制与病因分类

（一）发病机制

Virchow提出血栓形成三要素：血管壁因素（内皮细胞损伤）、血流淤滞、血液成分异常（血小板、凝血因子、抗凝蛋白、纤维蛋白溶解系统、炎症因子等）。因此，能够直接或间接影响上述三个基本环节的各种病理生理变化都可导致易栓症的发生，通常主要因凝血-抗凝血、纤溶-抗纤溶失衡引起的血液高凝状态所致[4]。

（二）病因分类

易栓症可分为遗传性和获得性。通过对家系和孪生子的配对研究表明，个体对VTE易感性的差异约60％归结于遗传因素[5]。遗传性易栓症常见于生理性抗凝蛋白——如抗凝血酶（antithrombin, AT）、蛋白C（protein C, PC）、蛋白S（protein S, PS）等基因突变导致蛋白抗凝血功能缺失，或促凝蛋白——如凝血因子V Leiden突变、凝血酶原G20210A基因突变等导致蛋白促凝功能增强，最终引起血栓栓塞[6]。获得性易栓症主要发生于各种获得性疾病或具有获得性危险因素的患者，因促凝蛋白水平升高、抗凝蛋白水平下降、改变了炎症/自身免疫机制等使血栓栓塞倾向增加。遗传性和获得性易栓因素存在交互作用，当二者同时存在时，血栓栓塞性疾病更易发生。易栓症常见病因见表1。

**表1:** 易栓症的分类与病因

遗传性易栓症	获得性危险因素	易栓症相关获得性疾病
抗凝血酶缺陷症^a^	年龄大于65岁^a^	抗磷脂综合征^a^
蛋白C缺陷症^a^	BMI大于30	活动性恶性肿瘤^a^
蛋白S缺陷症^a^	吸烟	骨髓增殖性肿瘤^a^
血栓调节蛋白缺陷^a^	多发性外伤^a^	肾病综合征^a^
*APOH*基因突变	大手术^a^	阵发性睡眠性血红蛋白尿^a^
肝素辅因子Ⅱ基因突变	骨折^a^	炎症性肠病
FⅧ水平升高^a^	脱水	系统性红斑狼疮
FⅨ水平升高^a^	妊娠/产褥期	系统性血管炎^a^
FⅪ水平升高^a^	下肢瘫痪或麻痹	急性心肌梗死
*F2*突变导致抗凝血酶抵抗^a^	肢体制动/长期卧床	急性卒中^a^
异常纤维蛋白原血症^a^	长途飞行	糖尿病
PAI-1水平升高	一些化疗药物	感染与炎症（结核、AIDS、胰腺炎）
血红蛋白病/地中海贫血^a^	脾切除/脾动脉栓塞	肝素诱导的血小板减少症^a^
高同型半胱氨酸血症^a^	中心静脉穿刺	Cushing综合征
*F5* Leiden突变^a^	人工材料（心瓣膜、留置导管等）^a^	布-加综合征^a^
*F2* G20210A突变^a^	输注血制品（红细胞、血小板）	血栓性微血管病（TTP、HUS）^a^
CHAPLE综合征^a^	止血治疗（抗纤溶、凝血因子制剂）	心力衰竭
克氏综合征	造血刺激因子（EPO、TPO等）	慢性肺病（呼吸衰竭、COPD）
其他罕见的遗传性易栓症	药物（糖皮质激素、避孕药、雌激素、睾酮治疗、抗精神病药物）	高黏滞血症（巨球蛋白血症、M蛋白血症）^a^

注：^a^ 表示静脉血栓栓塞（VTE）的高危险因素，否则为中低危险因素。本指南中高危险因素指文献所报道VTE的相对风险增加3倍以上，中低危险因素指文献所报道VTE的相对风险增加1～3倍；PAI-1：纤溶酶原激活物抑制剂-1；EPO：促红细胞生成素；TPO：促血小板生成素；TTP：血栓性血小板减少性紫癜；HUS：溶血性尿毒综合征；COPD：慢性阻塞性肺疾病；FⅧ、FⅨ、FⅪ分别为凝血因子Ⅷ、Ⅸ、Ⅺ

遗传性易栓症存在显著的种族差异。欧美人群以凝血因子功能增强为主，包括F5 Leiden和F2 G20210A突变，这2种突变在我国人群罕见；而我国和亚洲其他地区以抗凝蛋白缺陷为主，包括抗凝血酶缺陷症、蛋白C缺陷症、蛋白S缺陷症、血栓调节蛋白（thrombomodulin, TM）缺陷症等[7]–[8]。来自华中地区的易栓症分子遗传学研究显示，蛋白C抗凝系统基因缺陷最为常见，至少存在3种优势基因变异，是引起血栓形成常见的遗传危险因素：包括PROC p.Arg189Trp、PROC p.Lys192del和THBD c.-151G>T，杂合子在汉族健康人群的比例为0.8％～2.4％，发生VTE的风险增加2.5～6.4倍[9]–[11]。这些重现性基因变异的临床意义在我国其他地区和亚洲其他国家陆续得到进一步证实[12]–[15]。此外，持续性凝血因子Ⅷ（FⅧ）水平升高、非O血型等也是影响血栓形成的常见遗传因素。

常见的易栓症相关获得性疾病/获得性危险因素包括抗磷脂综合征、自身免疫性疾病、恶性肿瘤、急性卒中、慢性心肺疾病、慢性肾病、高龄、肥胖、手术、肢体制动或长期卧床、多发性外伤、骨折等[16]–[22]。

二、易栓症的诊断和病因筛查

（一）病史采集

1. 根据易栓症常见病因询问既往有无基础疾病：VTE病史、感染、手术、外伤、充血性心力衰竭、慢性呼吸系统疾病、自身免疫疾病、血液系统疾病及实体肿瘤等。

2. 用药史：了解患者有无口服避孕药、雌激素替代治疗、化疗、靶向药、免疫调节剂等。

3. 是否正在妊娠、近期分娩或剖宫产、既往有无不良妊娠史等。

4. 家族史：患者近亲有无VTE相关病史，父母有无近亲结婚、遗传性疾病等。

（二）实验室检查

1. 全血细胞分类与计数、网织红细胞计数及外周血涂片。

2. 尿常规、大便常规、肝功能、肾功能、球蛋白、血脂、乳酸脱氢酶、血型、血糖、同型半胱氨酸等。

3. 凝血指标：凝血酶原时间（PT），活化部分凝血活酶时间（APTT），凝血酶时间（TT），D-二聚体，纤维蛋白/纤维蛋白原降解产物（FDP），纤维蛋白原（Fg），内外源性凝血因子水平，血管性血友病因子（VWF）水平，抗凝血酶活性，蛋白C活性，蛋白S游离抗原。

4. 免疫指标：红细胞沉降率，C反应蛋白，狼疮抗凝物，抗心磷脂抗体，抗β_2_-糖蛋白Ⅰ（抗β_2_-GPⅠ）抗体，其他自身免疫抗体以及免疫球蛋白、补体水平。

5. 易栓症的高通量测序基因诊断【2B】：根据既往出凝血相关经典基因的研究以及新近基因组学对血栓形成新候选基因的探索，目前已知至少156种基因及其表达产物能够直接或间接影响血栓与止血，引起不同程度的易栓症。这些基因涉及凝血、抗凝血、纤溶、抗纤溶、血小板、血管内皮细胞、炎症反应等多个系统。建立同时涵盖这些基因的高通量测序技术能够全面和精准地诊断遗传性易栓症，是未来易栓症基因诊断的趋势[23]。

（三）VTE的临床诊断

应结合临床表现、D-二聚体、影像学检查进行临床诊断[24]。

1. 下肢深静脉血栓形成（deep vein thrombosis, DVT）：下肢不对称肿胀、疼痛和浅静脉曲张是下肢DVT的三大症状。根据下肢肿胀的平面可初步估计静脉血栓形成的部位。疼痛性质多为钝痛或胀痛。确诊DVT的方法主要是下肢静脉加压超声显像；静脉造影为有创性检查，只用于高度疑诊但超声检查阴性者。

2. 肺栓塞（pulmonary embolism, PE）：多为急性发病，临床表现多样、缺乏特异性，包括胸痛、咯血、呼吸困难、气促、心悸、晕厥等，严重时可发生低血压休克甚至猝死。三分之一的患者可因右房压力高和卵圆孔再开放，产生右向左分流的矛盾性栓塞。慢性PE可形成肺动脉高压和肺心病。确诊通常应用计算机断层扫描肺动脉造影（CTPA），特殊情况下可用核素肺通气/灌注显像、磁共振肺动脉造影替代。

3. 颅内静脉血栓形成（cerebral venous thrombosis, CVT）：根据急性、亚急性或慢性起病，血栓形成的部位、性质、范围以及继发性脑损害的程度等，临床症状不一、表现多样。常出现头痛、视力障碍、视乳头水肿、呕吐等颅内高压表现，严重时出现意识障碍甚至脑疝危及生命。部分患者可有局灶性脑损害、痫性发作以及搏动性耳鸣。约30％的慢性期患者可有不同程度的认知障碍。对疑似CVT的患者，计算机断层扫描（CT）/计算机断层扫描静脉造影（CTV）和磁共振成像（MRI）/磁共振静脉造影（MRV）为主要的检查方法。前者快捷便利，后者能准确诊断大多数CVT，可作为无创性随访检查手段。

4. 门静脉血栓形成（portal vein thrombosis, PVT）：部分PVT患者无血栓相关症状。最常见的临床表现为腹痛和消化道出血（呕血、黑便或鲜血便）；可出现胆管病变或肠缺血症状（发热、黄疸、皮肤瘙痒、胆绞痛、腹痛、腹水等）；门脉高压时可出现食管胃底静脉曲张和脾肿大。腹部影像学检查可先行多普勒超声筛查，再行CT/CTV或MRI/MRV确诊。

5. 肠系膜静脉血栓形成（mesenteric venous thrombosis, MVT）：急性期表现为持续数小时至数周不等、与腹部体征不相称的脐周绞痛。与肠系膜缺血相比，这种绞痛发作不是突然发生，呈持续性钝痛，可伴有阵发性加重，可有恶心和呕吐。亚急性期或慢性期疼痛逐渐减轻。腹部检查可能有腹部膨隆等体征，大便隐血可能呈阳性。患者通常无腹膜炎征象，但肠扩张进展时可出现肠缺血坏死，肠鸣音消失，并出现压痛反跳痛等。诊断MVT最准确的方式是MRV，紧急情况下推荐CTV作为初始筛查。

（四）易栓症病因筛查的指征【1C】

1. 小于50岁的VTE或无动脉粥样硬化危险因素的发病年龄较低的动脉血栓形成患者；

2. 无明显诱因的特发性VTE患者；

3. 有明确家族史的VTE患者；

4. 复发性VTE患者；

5. 少见部位的VTE（脾静脉、颅内静脉、门静脉、肠系膜静脉、肝静脉、肾静脉、上肢深静脉）或多部位、累及范围广的VTE患者；

6. 标准方案抗栓过程中出现皮肤坏死、血栓加重或复发的患者；

7. 新生儿暴发性紫癜；

8. 不明原因的多次病理性妊娠（习惯性流产、胎儿发育停滞、死胎、子痫前期、胎盘早剥等）；

9. 有VTE病史或家族史者，拟行大型手术、妊娠、使用性激素类药物及频繁长途飞行前可进行筛查。

（五）易栓症病因筛查的时机[25]

1. VTE急性期和抗凝用药会影响抗凝蛋白水平的检测（表2），此时抗凝血酶、蛋白C、蛋白S的检测结果仅供排除性参考。因此，抗凝蛋白水平检测应在血栓急性期后、停止抗凝治疗2周以上进行【1A】。

**表2:** 易栓症实验室筛查的干扰因素

检测项目	VTE急性期	肝素类	VKA	DOAC
抗凝血酶活性（发色底物法）	-或↓	↓	-	抗FⅩa活性↑（FⅩa抑制剂）；抗FⅡa活性↑（FⅡa抑制剂）
蛋白C活性（发色底物法）	-或↓	-	↓	-
蛋白S活性（凝固法）	-或↓	-	↓	-或↑
游离蛋白S抗原	-或↓	-	↓	-
狼疮抗凝物	-	-或假阳性	-或假阳性	-或假阳性
抗心磷脂抗体	-	-	-	-
抗β_2_-GPⅠ	-	-	-	-
FⅧ活性（凝固法）	↑	↓	-	-或↓
活化蛋白C抵抗	-	-	-	-或假阴性

注：-：对实验结果不会产生干扰；VTE：静脉血栓栓塞症；VKA：维生素K拮抗剂（通常指华法林）；DOAC：直接作用口服抗凝药物；FⅧ：凝血因子Ⅷ；FⅩa：凝血因子Ⅹa；FⅡa：凝血因子Ⅱa

2. 抗凝蛋白活性水平还受其他获得性因素影响出现一过性降低（表3），因此不应该仅凭一次实验室检测结果确诊遗传性抗凝蛋白缺陷【1A】。

**表3:** 抗凝血酶、蛋白S、蛋白C获得性缺陷的原因

抗凝蛋白	原因
抗凝血酶	血栓形成急性期；肝硬化；肾病综合征；蛋白丢失性肠病；体外膜肺氧合；左旋门冬酰胺酶/培门冬酶；肝素类药物治疗；严重烧伤；妊娠高血压、子痫前期和子痫；DIC
蛋白C	血栓形成急性期；肝硬化；脓毒症；DIC；维生素K缺乏或口服华法林；新生儿时期；自身抗体
蛋白S	血栓形成急性期；肝硬化；妊娠；产褥期；避孕药；雌激素替代治疗；DIC；维生素K缺乏或口服华法林；新生儿时期；自身抗体；血浆置换；炎症状态；AIDS

注：DIC：弥散性血管内凝血；AIDS：获得性免疫缺陷综合征

3. LA的检测应在抗凝治疗前或停用口服抗凝药至少1周后进行，阳性结果应在12周后复测排除一过性异常【1A】。

4. 基因检测可在任意时间点进行，高通量测序的阳性结果需再次采集样本用一代测序验证【2A】。

（六）易栓症病因筛查流程

易栓症不是单一疾病，涉及众多病因和危险因素，全面筛查项目多、费用高，建议依据患者年龄、性别、病史、潜在病因发生率的高低、常规检查结果的提示等确定筛查方向和顺序，通常情况下可按照图1流程进行易栓症病因筛查【2C】。需要注意血栓形成是多因素共同作用，患者可同时存在多种易栓症危险因素，如多基因复合型突变的遗传性易栓症、恶性肿瘤合并遗传性易栓症、恶性肿瘤继发抗磷脂综合征、风湿免疫疾病合并妊娠等。因此，筛查时不能满足和局限于第一发现，尤其是筛查出较弱危险因素而不能解释易栓症严重程度时，需进一步检查病因。目前仍有部分患者全面筛查也不能完全明确血栓病因，有待继续深入探索。

**图1:**
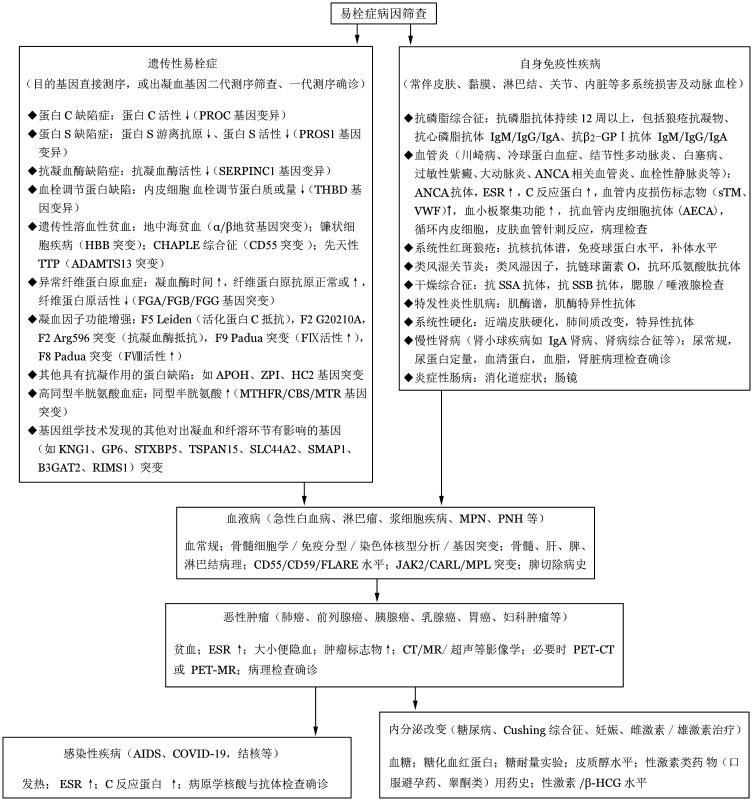
易栓症病因筛查流程 ESR：红细胞沉降率；sTM：可溶性血栓调节蛋白；VWF：血管性血友病因子；MPN：骨髓增殖性肿瘤；PNH：阵发性睡眠性血红蛋白尿症；CT：计算机断层扫描；MR：磁共振；PET：正电子发射断层扫描；COVID-19：2019冠状病毒病；FⅧ：凝血因子Ⅷ；FⅨ：凝血因子Ⅸ；β-HGG：绒毛膜促性腺激素

三、易栓症的治疗和长期管理

遗传性易栓症目前尚无根治方法，治疗主要针对血栓栓塞症进行抗栓治疗；除了抗栓治疗以外,获得性易栓症应积极治疗原发疾病，祛除和纠正诱发因素[26]–[27]。

VTE的治疗包括药物抗凝（口服或胃肠外用药）、溶栓（系统溶栓或导管接触溶栓）、介入治疗和手术治疗；预防措施包括基础预防（下肢活动、避免脱水等）、物理预防（腔静脉滤器植入、分级弹力袜、足底静脉泵、间歇性充气加压装置等）和药物抗凝预防[28]–[29]。根据血栓形成不同部位，具体抗栓用药细则可参考其他专科指南和共识。

易栓症长期管理的主要目标为预防血栓事件复发，应遵循以下基本原则（图2）：

**图2:**
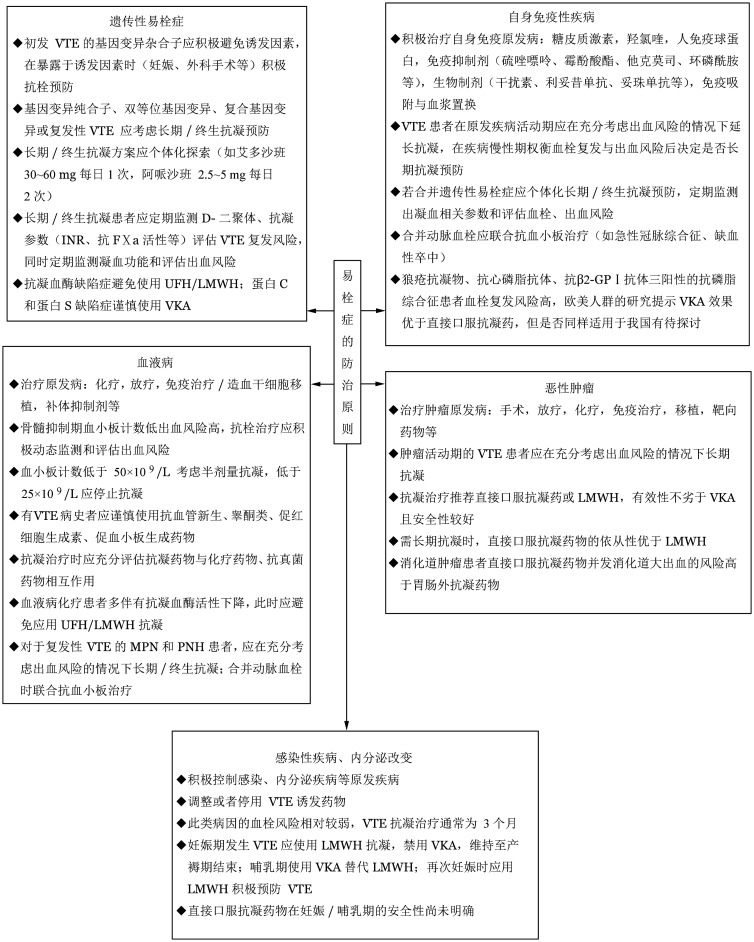
易栓症的防治原则 VTE：静脉血栓栓塞症；INR：凝血酶原时间的国际标准化比值；UFH：普通肝素；LMWH：低分子量肝素；MPN：骨髓增殖性肿瘤；PNH：阵发性睡眠性血红蛋白尿症；VKA：维生素K拮抗剂；FⅩa：凝血因子Ⅹa

（1）建议进行多学科评估，结合患者的易栓症病因、年龄、性别、合并症和依从性，确定抗凝药物种类、剂量、用药途径、抗凝时程，探索个体化防治方案【1A】，常用的抗凝药物见表4。

**表4:** 常用的抗凝药物

抗凝药物	半衰期	主要作用机制	成人常规VTE治疗剂量	成人常规VTE预防剂量
阿加曲班	15～30 min	直接抑制凝血酶	40～60 mg 24 h持续泵入	10 mg 3 h静脉滴注，每12 h 1次
艾多沙班	10～14 h	直接抑制FⅩa	60 mg每日1次，口服	30～60 mg每日1次，口服
阿哌沙班	12 h	直接抑制FⅩa	5～10 mg每日2次，口服	2.5～5 mg每日2次，口服
利伐沙班	5～13 h	直接抑制FⅩa	15 mg每日2次，口服	10～20 mg每日1次，口服
达比加群	12～14 h	直接抑制凝血酶	110～150 mg每日2次，口服	75～110 mg每日2次，口服
依诺肝素	5～7 h	增强抗凝血酶活性	100 IU/kg每12 h 1次，皮下注射	100 IU/kg每日1次，皮下注射
华法林	36～42 h	维生素K拮抗剂	维持INR 2～3	维持INR 2～3

注：VTE：静脉血栓栓塞症；FⅩa：凝血因子Ⅹa；INR：凝血酶原时间的国际标准化比值

（2）VTE急性期治疗结束后，对于是否需要延长、长期/终生抗凝预防，应充分评估患者血栓复发风险和出血可能性，权衡风险和获益【1A】；如需要延长、长期/终生抗凝，应定期、规律对血常规、肝肾功能、凝血功能、D-二聚体、抗凝血参数（如抗FⅩa活性）、血栓影像学进行监测，评估预防效果和出血风险【1C】。

（3）对于尚未发生血栓事件的易栓症，只需进行基础预防，通常无须采取预防性抗凝，但应避免VTE诱发因素【1C】；在暴露于危险因素时，如高龄、长途飞行、大手术、使用特殊药物或妊娠，应预防性药物抗凝，存在抗凝禁忌时考虑采用物理预防【2C】。

（4）对于血栓初次发生的易栓症患者，抗凝治疗3～6个月，同时积极祛除诱发因素和纠正病因【1C】；若病因暂时无法祛除，应延长抗凝（如6～12个月），之后再次评估【1C】。

（5）对于血栓反复发作且无明显出血风险的易栓症患者应进行长期/终生抗凝【2C】；小剂量艾多沙班（30 mg每日1次）、阿哌沙班（2.5 mg每日2次）可作为预防性抗凝的初始选择【1B】，这些药物可在不增加大出血风险的情况下显著降低VTE复发率。

（6）某些特定的易栓症在选择抗凝药物时应予特殊注意：抗凝血酶缺陷患者使用普通肝素（unfractioned heparin, UFH）或低分子量肝素（low molecular weight heparin, LMWH）效果不佳【1C】，胃肠外抗凝可选择阿加曲班等凝血酶直接抑制剂【2C】；蛋白C和蛋白S缺陷患者不能使用华法林等香豆类抗凝剂作为初始抗凝治疗，因可引起血栓倾向加重、皮肤坏死【1A】；对于同时存在出血风险或围手术期预防的患者，建议使用阿加曲班等半衰期短的抗凝药物【2C】。

（7）抗凝治疗的主要不良反应是出血，严重者可致残甚至危及生命，相对于欧美人群而言，我国人群普遍属于较“低凝”或“易出血”体质，需警惕出血风险【1C】。出血评估需考虑的因素见表5。

**表5:** 易栓症患者抗凝治疗中发生大出血的高危因素

内科因素
年龄≥75岁
活动性出血或大出血病史
遗传性或获得性出血性疾病
肝功能不全（INR>1.5）
严重肾功能不全（GFR<30 ml/min/m^2^）
急性脑卒中
未控制的高血压
血小板减少症（PLT<50×10^9^/L）
活动性消化性溃疡
合并使用抗凝/抗血小板/溶栓/NSAID药
外科因素
腰穿、硬膜外或椎管内麻醉操作12 h内
男性腹部恶性肿瘤复杂性手术且术前HGB低于130 g/L
心脏手术伴有以下因素之一：正在使用阿司匹林；术前3 d内使用氯吡格雷；BMI大于25、≥5个支架的非择期手术；高龄、肾功能不全、体外循环时间长的非搭桥手术
伴有败血症、胰漏或前哨肠袢出血的胰十二指肠切除术
原发性肝癌或多叶切除、伴随肝外器官切除的肝脏手术
神经外科手术、脊柱手术
游离皮瓣重建术
肺切除术或扩大切除手术

注：大出血：血液动力学不稳定（收缩压<90 mmHg或收缩压下降≥40 mmHg）、重要脏器（颅内、椎管内、眼内、腹膜后、关节内、心包、肌肉内、气道主干）出血而需外科手术、1 d内血红蛋白下降≥20 g/L、需输注压积红细胞至少2 U、致死性的出血事件。NSAID：非甾体抗炎药；INR：凝血酶原时间的国际标准化比值；GFR：肾小球滤过率；BMI：身体质量指数

（8）对于危及生命的VTE事件（伴有休克的肺栓塞、严重颅内压升高的静脉窦血栓形成、肠坏死风险的门静脉/肠系膜静脉血栓等），高出血风险的抗凝绝对禁忌可视为相对禁忌【2A】。

（9）抗凝治疗过程中VTE加重或抗凝预防过程中VTE复发，应考虑以下因素：评估VTE是否确实加重或复发；非血栓栓塞（如癌栓、细菌等栓子）；确认患者用药的依从性；发生肝素诱导的血小板减少症（正在使用肝素或低分子量肝素）；用药种类是否合适（如抗凝血酶缺陷症使用低分子量肝素效果不佳）；用药方案是否合适（如标准剂量利伐沙班需随餐口服）；药物相互作用降低抗凝药物浓度；慢性腹泻导致脱水以及影响口服药物吸；存在附加的易栓症因素因而高凝状态较重（如合并恶性肿瘤或多种易栓症相关基因突变），此时可考虑增加用药剂量（如低分子量肝素每次增加1/4至1/3剂量）或更换抗凝药物（如直接口服抗凝药物替换为华法林）【2C】。

（10）对于增加抗凝剂量甚至联合溶栓的情况下VTE加重或复发，应考虑血管壁因素的易栓症（如系统性血管炎）；此时再增加抗凝剂量只会增加出血风险却不能有效控制血栓形成，应加用糖皮质激素、免疫抑制剂等药物联合抗栓【1B】。
